# Thermal and Photocatalytic Performance of Unsaturated Polyester Resins Modified with TiO_2_ Nanoparticles as Panel Bodies for Vehicles

**DOI:** 10.3390/polym13132036

**Published:** 2021-06-22

**Authors:** Miren Blanco, Cristina Monteserín, Nerea Uranga, Estíbaliz Gómez, Estíbaliz Aranzabe, Jose Ignacio García

**Affiliations:** 1 Surface Chemistry and Nanotechnology Unit, Tekniker-BRTA, Calle Iñaki Goenaga 5, Eibar, 20600 Gipuzkoa, Spain; cristina.monteserin@tekniker.es (C.M.); nerea.uranga@tekniker.es (N.U.); estibaliz.gomez@tekniker.es (E.G.); estibaliz.aranzabe@tekniker.es (E.A.); 2 Tecnove, Avenida de Alcazar 6, Herencia, 13640 Ciudad Real, Spain

**Keywords:** total solar reflectance (TSR) photocatalysis, TiO_2_ nanoparticles, thermal performance, unsaturated polyester resin, kinetics

## Abstract

The transport sector is the fastest growing contributor to climate emissions and experiences the highest growth in energy use. This study explores the use of TiO_2_ nanoparticles for obtaining photocatalytic nanocomposites with improved infrared reflectance properties. The nanocomposites were prepared by dispersing 0–20 wt% of TiO_2_ nanoparticles in an unsaturated polyester resin. The effect of TiO_2_ on the curing kinetics was studied by differential scanning calorimetry, showing a significant delay of the curing reactions. The thermal reflectance of the modified resins was characterized by UV-Vis-NIR spectrophotometry, measuring total solar reflectance (TSR). The TiO_2_ greatly increased the TSR of the resin, due to the reflectance properties of the nanoparticles and the change in color of the modified resin. These nanocomposites reflect a significant part of near-infrared radiation, which can contribute to a reduction of the use of heating, ventilation, and air conditioning. Moreover, the photocatalytic effect of the TiO_2_ modified nanocomposites was studied by monitoring the degradation of an organic model contaminant in an aqueous medium under UV light, and the reusability of the nanocomposites was studied with 5 cycles. The developed nanocomposites are proposed as a solution for reducing global warming and pollutant emissions.

## 1. Introduction

Transportation is an essential part of human activity which supports socio-economic activities. Efficient transportation systems provide access to key economic inputs such as resources, knowledge, and technology; reduce the barriers to free movement of goods and persons, and increase the market for goods and services [1]. The transport of perishable food items requires temperature-controlled and conditioned freight shipment. Studies indicate that the global refrigerated transport market was estimated at USD 14.8 billion in 2019 and is projected to grow at a compound annual growth rate (CAGR) of 5.9% from 2019 to reach 23.1 billion by the year 2027 [2]. This represents a grave situation in view of energy demand to sustain these refrigerated vehicles and in the production of greenhouse gases. Transportation is responsible for almost a quarter of Europe’s greenhouse gas emissions, road transportation being the biggest emitter and is the main cause of air pollution in cities [3]. Nearly 87% of the world’s population live in countries where the ambient levels of air pollution exceed the guidelines set by the World Health Organization (WHO) [4]. Recently, the International Agency for Research on Cancer (IARC) has classified urban air pollution as carcinogenic to humans [5].

**Figure 1:**
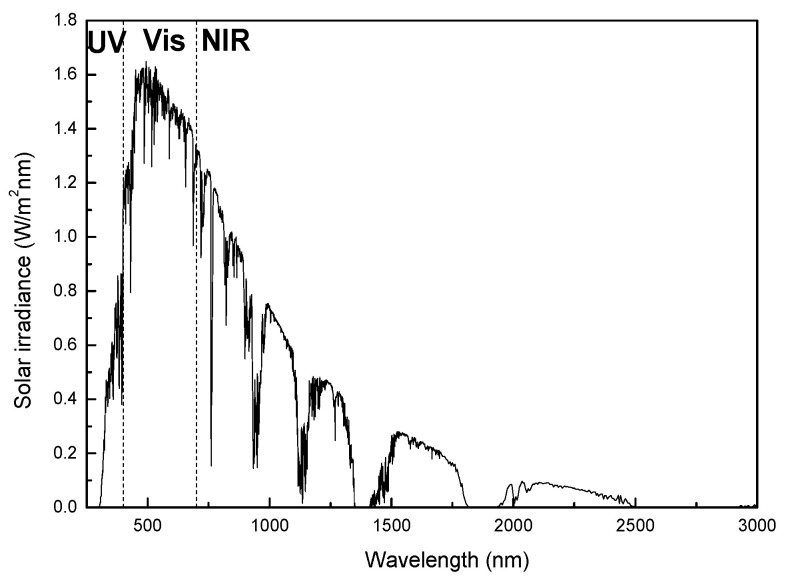
Spectrum of solar irradiance, according to ASTM G173-03.

To avoid the increased environmental impact of refrigerated vehicles, it would be of great interest to reduce their energy consumption. Insulated panels in refrigerated vehicles remain the channel through which heat infiltration into the cooling chamber is controlled [6]. Insulated panels are three layered plane structures where an insulation, typically polystyrene or polyurethane, is sandwiched between two thin skin layers of polymeric sheets bonded to each side, usually a glass fiber/resin composite. Different energy reduction techniques have been explored for these vehicles, mainly focused on the use of different insulation materials with low thermal conductivity, such as porous polymer foams [7], aerogels or xerogels [8,9], and the optimization of insulation thickness [6]. However, some of these insulation materials are costly and difficult to produce. Other strategies explore the use of phase change materials (PCMs) with a high thermal storage density for thermal energy storage (TES), which offers enhancing the energy efficiency of cold storage and transportation systems [10,11,12], but the effect of PCM is conventionally short in time. The modification of thermo-optical properties of the external glass fiber/resin composite in the panels can be an interesting alternative. It has been reported that the thermo-optical properties of the external surface of the vehicle bodywork can account for up to 40% of the energy consumption of the refrigerated unit [13]. When sun electromagnetic radiation strikes a surface, it is partially absorbed and transferred into heat causing a temperature rise in the surface. As reported in Figure 1 [14], according to the tabulated data in ASTM G173-03 of the solar irradiance on the earth surface at air mass 1.5, the typical spectral distribution with wavelengths ranging from 200 to 2500 µm can be divided into three regions: 5% of solar radiation consists of ultraviolet range (UV, <400 nm); 43% consists of visible range (VIS, 400–700 nm), which is responsible for color perception; 52% consists of near-infrared light (NIR, >700 nm), which is responsible for the heat build-up. A proper design of the external surface of the refrigerated trailer insulation walls can improve the thermal radiation protection of the system.

In the field of air cleaning technologies, heterogeneous photocatalysis is a well-established technology for treating environmental pollutants. Photocatalysis refers to the photogeneration of strong oxidizing and reducing agents at the surface of the catalyst that act to destroy pollutants, especially organic pollutants, obtaining a complete mineralization of many organic and inorganic pollutants present in air and water [15]. Different catalysts have been shown to be effective for the heterogeneous catalytic products, such as metal oxides, supporting metals or metal-free materials. However, they present several drawbacks that limit their wide application, such as metal leaching to the environment, reduced efficiency, durability, stability and cost [16,17,18,19]. Between photocatalysts, TiO_2_ nanoparticles are one of the most promising and widespread photocatalysts as: (1) the nanometric size gives them a large specific area, increasing their reactivity; (2) they are commercially viable at large scale, abundant and thus inexpensive, and (3) TiO_2_ is chemically stable and possesses a high capability to catalyze degradation processes via the disruption of molecular bonds. Moreover, TiO_2_ photocatalytic activity even remains immobilized in other materials. Immobilized photocatalytic materials allow for applications in air and water treatment, as this prevents entraining nanometer sized particulates in the air or water stream, which could pose environmental and health risks [16]. Furthermore, a well-immobilized catalyst will suffer less physical deterioration over time from ambient conditions (flow, mechanical abrasion, etc.), perform better over the long term and allow easier recovery of catalyst from the stream and its reuse [17]. The incorporation of TiO_2_ in the outer layers of the insulated panel could be an interesting option to allow the vehicles to reduce the pollutants generated during their own use. Moreover, TiO_2_ is used as white reflective pigment in development of reflective coatings because of its effective light scattering properties [20,21].

In this context, this study explores the use of TiO_2_ for obtaining nanocomposites with improved infrared reflectance properties and air detoxification properties. The nanocomposite series was prepared by dispersing 0–20 wt% of TiO_2_ in an unsaturated polyester resin. Unsaturated polyester resins are widely used as the matrix resin for polymeric composites because of their relatively low cost, good balance of properties, and adaptability to many fabrication processes. The effect of TiO_2_ nanoparticles on the curing kinetic of the orthophthalic resin has been studied by differential scanning calorimetry, showing that TiO_2_ nanoparticles delay and even avoid the curing of the resin at low temperatures. The reflection capability of the resins and composites were characterized by UV-Vis-NIR spectrophotometry, measuring total solar reflectance (TSR) and thermal performance of the materials. Moreover, the photocatalytic effect of the TiO_2_ modified resins under UV light and their reusability has been studied regarding the degradation of an organic model contaminant dissolved in water. The developed nanocomposites are proposed as a solution for reducing global warming and pollutant emissions. 

## 2. Materials and Methods

### 2.1. Materials

The resin employed in this study was a pre-accelerated orthophthalic unsaturated polyester resin in styrene (39–42 wt% styrene content), supplied by Polynt Composites with the commercial name DISTITRON^®^ 5119 E1SX20Q. To initiate the reaction, a source of free radicals is needed, by means of heat or a catalytic system. Organic peroxides (initiators) are commonly used as the source of free radicals. A 2 wt% amount of methyl ethyl ketone peroxide (MEKP) initiator was used, dissolved in diisobutyl phthalate, from Akzo Nobel with the commercial name BUTANOX^®^ M50. Titanium dioxide of analytical grade of purity (Aeroxide^®^ P25 TiO_2_, <25 nm) was obtained from Evonik Corporation (Essen, Germany). For testing the photocatalytic properties of developed nanocomposites, a model organic pollutant, a commercial diazo reactive dye containing two vinyl sulfones as reactive groups, Remazol Black B (C.I. Reactive Black 5) was employed. This dye has the empirical formula C_26_H_21_N_5_Na_4_O_19_S_6_ and a molecular weight of 991.8 g/mol and was purchased from Acros Organics (Morris, NJ, USA). All chemicals are analytical grade substances and have been used without further purification. 

### 2.2. Sample Preparation

TiO_2_ nanoparticles were dispersed in the unsaturated polyester resin (UP) by 30 min of mechanical mixing with a Heidolph^®^ RZR-1 overhead Stirrer 230 V 50 Hz. The temperature of the mixture during mixing was kept cold by using an ice bath to avoid styrene evaporation. The nanoparticle modified resin and the curing agent were also mixed thoroughly by mechanical mixing, obtaining mixtures with 0–20 wt% of nanoparticles. To prepare the specimens required for the different tests, all mixtures were poured into glass molds and thereafter, neat systems were cured 24 h at room temperature and modified systems 6 h at 80 °C. Finally, they were post-cured for 2 h at 120 °C. 

### 2.3. Characterization Techniques

The influence of the addition of TiO_2_ nanoparticles in the reaction between the unsaturated resin and the catalyst was analyzed by differential scanning calorimetry (DSC). DSC measurements were performed in a DSC1 module from Mettler-Toledo (Gießen, Germany) equipped with an intracooler and previously calibrated with high-purity indium and zinc standards. All of the measurements were conducted in a dry atmosphere under a constant nitrogen flow of 50 mL/min, working with samples between 7 and 10 mg. Runs at a constant heating rate of 10 °C/min were performed in a temperature range from 30 to 225 °C. The glass transition temperature of the fully cured material, Tg^∞^, was determined with a second dynamic scan at 20 °C/min in the same temperature range. Tg^∞^ was taken as the middle point of the endothermic shift. Moreover, kinetic study was also performed with the DSC equipment working isothermally at temperatures ranging from 30 to 70 °C for 3 h. All the samples were then cooled to room temperature and subjected to a dynamic DSC scan from 30 to 225 °C at 10 °C/min to determine the residual heat of reaction. Field emission scanning electron microscopy (FE-SEM) was employed to analyze the morphology and topography of unmodified and modified systems, by using a Carl Zeiss SMT Ultra Gemini-II microscope (Carl Zeiss, Thornwood, NY, USA). Samples were analyzed without being coated. 

To determine the reflection capability of the nanoparticles, the total solar reflectance (TSR) values of the resins with different contents of nanoparticles were measured in a Perkin Elmer Lambda 950 UV/Vis/NIR System in the wavelength range from 300 to 2500 nm according to ASTM G173 and using poly-tetrafluoroethylene (PTFE) as the white standard. TSR is the percentage of irradiated energy that is reflected by an object. Thermal conductivity of the developed materials was measured by using a thermal conductivity analyzer TCi-2A (TCi Mathis Instruments). The dimensions of the measured samples were discs, 5 cm in diameter and 2 cm thick. Measurements were carried out at 23 °C. Five specimens were measured for each material type.

**Figure 2:**
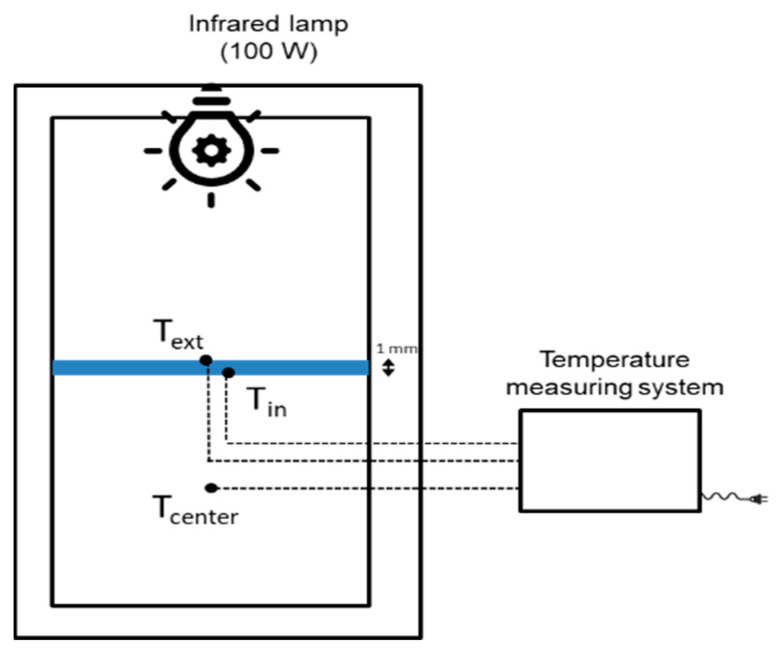
Schematic diagram of thermal performance test setup.

Thermal performance of composites in terms of heat transfer and surface temperatures of the specimens exposed to infrared light were evaluated by using the heating setup shown in Figure 2. 

The heating setup mainly consisted of a 100 W infrared lamp mounted at the top, a test box made of insulated polyurethane boards and a multichannel temperature measuring system. To study the thermal performance and the relative cooling effect of the materials with nanoparticles, the specimens with size of 200 × 200 × 1 mm^3^ were placed at the central position, allowing an empty chamber in the bottom part of the box simulating the indoor environment in a vehicle. The distance between the infrared lamp and the panel was 45 cm. The temperature was recorded by thermocouples placed at: (1) the center position of the chamber at the bottom part of the box to record the temperature variation during the process of heating and cooling (T_center_), (2) the upper surface of the specimen (T_ext_), and (3) the bottom surface of the specimen (T_in_). The duration of the heating step was 20 min. Thereafter, the infrared lamp was switched off, and the specimen was naturally cooled down for 30 min.

To determine the photocatalytic performance of the TiO_2_ modified materials, the capacity of the material for decomposition of an organic model pollutant, Remazol Black B [22], was studied. A stock solution was prepared with 3 mg/L of dye in distilled water and pH was adjusted to pH = 3. Samples of 1 mm thick materials were cut and immersed into the solution having a total surface of material in contact with the solution of 1.5 cm^2^/mL. The systems were irradiated for 23 h by two UV lamps with an emission maximum at 365 nm (UV lamp Vl-6-L, 145 × 48 mm^2^, 6 W from Vilber Lourmat, Collégien, France) and an intensity at 15 cm of 700 µW/cm^2^. The distance between the lamps and the solutions during the test was 5 cm. The photocatalytic process was monitored by following the color removal of the Remazol Black B solution by using UV-visible spectroscopy. Adsorption of dye on the surface of photocatalyst is an important parameter in heterogeneous photocatalysis therefore similar experiments were performed without UV radiation. The color removal was calculated based on the intensity decrease of the dye absorbance at 597 nm with respect to the irradiation time. UV-visible spectra at the wavelength range 380–1000 nm was recorded with a Perkin Elmer (Waltham, MA, USA) Lambda 950 spectrophotometer. Reusability of non-modified and modified systems was tested by measuring their adsorption and photocatalytic activity during five cycles. After each cycle, the materials were placed again in fresh Remazol Black B solution and tested, without any cleaning treatment. 

## 3. Results

### 3.1. Curing Kinetics and Morphology of Developed Composites

Before making any physical analysis on such materials, it is important to characterize the curing kinetics. The effect of TiO_2_ nanoparticles on the curing kinetic of the unsaturated polyester resin was studied by differential scanning calorimetry, in dynamic and isothermal modes. 

**Figure 3:**
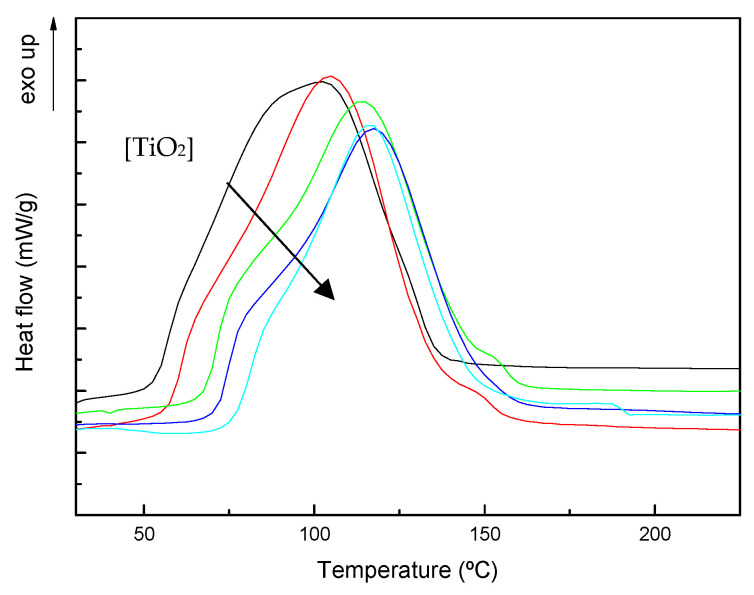
Non-isothermal DSC thermograms for (—) neat UP and systems with (—) 5, (—) 10, (—) 15 and (—) 20 wt% TiO_2_.

Dynamic DSC scans of neat unsaturated polyester resin and TiO_2_ modified systems are represented in Figure 3. All dynamic scans have a similar shape, indicating a similar cure reaction mechanism, with a main exothermic DSC peak (T_p2_) with a significant shoulder at lower temperatures (T_p1_). The first shoulder can be attributed to the polymerization initiated by the decomposition of MEKP caused by the presence of a promoter in the pre-accelerated resin, and the second one to the polymerization [23]. Free radicals formed during MEKP initiator decomposition trigger the polymerization, linking adjacent unsaturated polyester units and forming primary polymer chains. The polymer chains will grow and crosslink with styrene or the unsaturated polyester, according to two main reactive processes: styrene-polyester copolymerization and styrene homopolymerization. At temperatures around 150 °C a shoulder can be observed in some thermograms, which can be ascribed to the homopolymerization of polyester [24,25]. At high temperatures, polyester chains possess enough energy to override steric hindrances.

Table 1 gathers the main parameters obtained from dynamic scans, the exothermic peak temperatures T_p1_ and T_p2_, the temperature at which reaction starts (T_onset_), and heat reaction values (ΔH_T_). The presence of TiO_2_ nanoparticles delays the temperature at which reaction starts, and also T_p1_ and T_p2_ values, indicating a significant delay of the reaction kinetics. This could be ascribed to the increase of the initial viscosity of the mixture due to the addition of the nanoparticles. Interaction of the -OH groups on TiO_2_ surface with the carbonyl (C=O) of the ester group of the resin, already suggested for other systems [26,27], have not been observed by Fourier transform infrared spectroscopy (FTIR). The heat reaction values are slightly higher for the system with 5 wt% TiO_2_, but decrease for systems with higher nanoparticles contents due to the dilution effect due to the presence of the nanoparticles. However, values fall in the range of 230–330 J/g and similar results have been found in the literature for unsaturated polyester resins [21,28,29]. The Tg^∞^ values obtained in the subsequent scans, also collected in Table 1, are smaller for systems with higher TiO_2_ content indicating a small steric hindrance effect due to the presence of the high number of nanoparticles.

**Figure 4:**
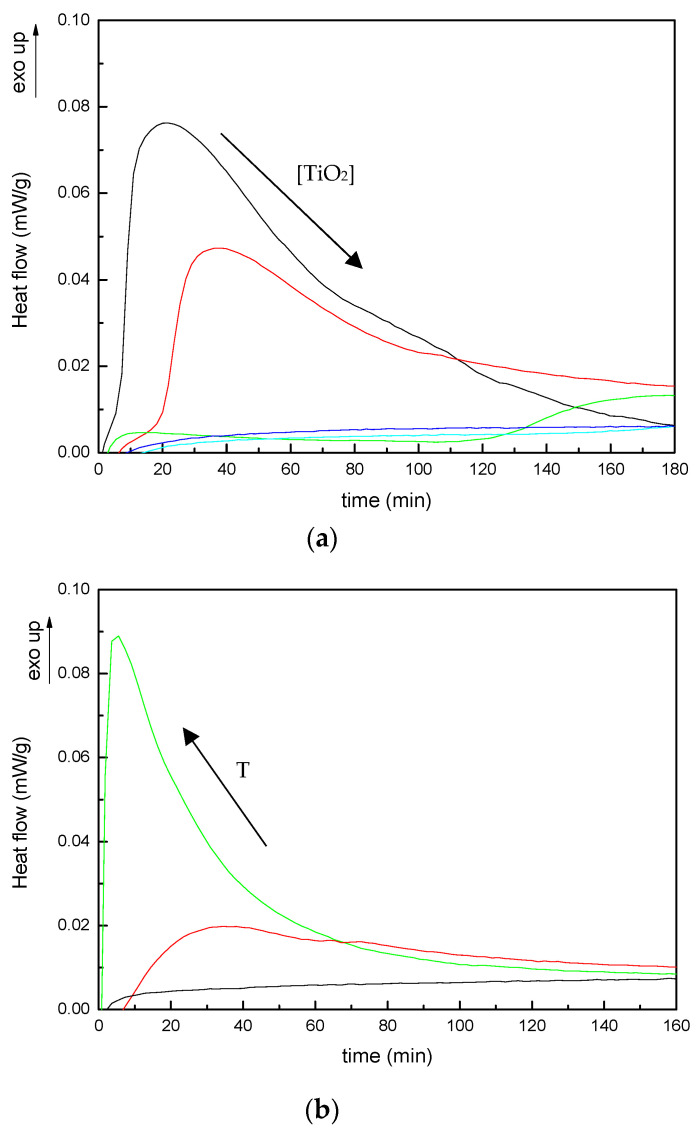
Isothermal DSC thermograms for: (**a**) (—) neat UP and systems with (—) 5, (—) 10, (—) 15 and (—) 20 wt% TiO_2_ at 30 °C; (**b**) UP system modified with 20 wt% TiO_2_ cured at: (—) 30 °C, (—) 50 °C and (—) 70 °C.

Polymerization kinetics of these systems was also studied at different isothermal temperatures ranging from 30 to 70 °C. Figure 4a shows the isothermal thermograms of the neat and modified systems at T = 30 °C. The reaction rate for neat system shows the typical behavior of free radical systems. Initially, the reaction rate increases with time, shows a maximum for time greater than zero, and then decreases as function of time. The addition of TiO_2_ nanoparticles to the unsaturated polyester resin does not modify this typical behavior for the system with 5 wt% TiO_2_, but for systems with higher contents of nanoparticles, the time at which the heat flow curve reaches a maximum and the time at which the reaction starts are greatly delayed and the reactions is not clearly observed. For systems with 15 and 20 wt% TiO_2_, the presence of nanoparticles almost completely hindered the reaction at this temperature. As has been commented before, an increase in the viscosity due to the presence of the nanoparticles can reduce the number of collisions between reactants at a similar temperature and thus reduce the reaction rate. Figure 4b shows the isothermal thermograms of 20 wt% TiO_2_ loaded nanocomposites performed at different temperatures. The reaction rate increases clearly with the increase in temperature.

**Figure 5:**
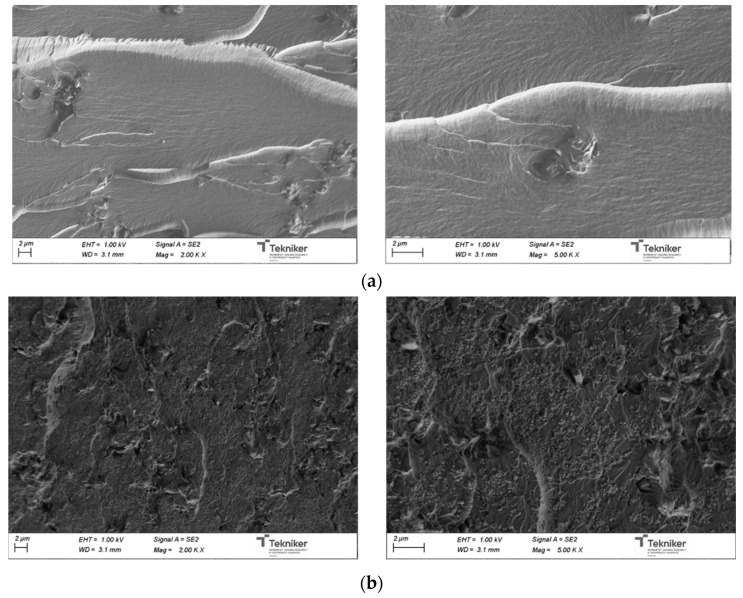
SEM images of (**a**) neat UP and (**b**) 20 wt%TiO2 modified UP composites.

Considering the kinetic analysis, the curing cycle was selected and composites with 0–20 wt% TiO_2_ were prepared. The morphology of the obtained materials was investigated by means of FE-SEM on the fracture surface of the cured systems. In Figure 5, the FE-SEM images of the neat unsaturated polyester resin and the resin modified with 20 wt% TiO_2_ nanoparticles are shown. A clear difference in fractured surface topography was observed for both systems due to the presence of TiO_2_ nanoparticles in the modified system. Moreover, a homogeneous distribution of nanoparticles is observed, with a low degree of particle agglomeration, which will be essential for the catalytic activity of the nanocomposites [30]. 

### 3.2. Thermal Reflectance Properties

**Figure 6:**
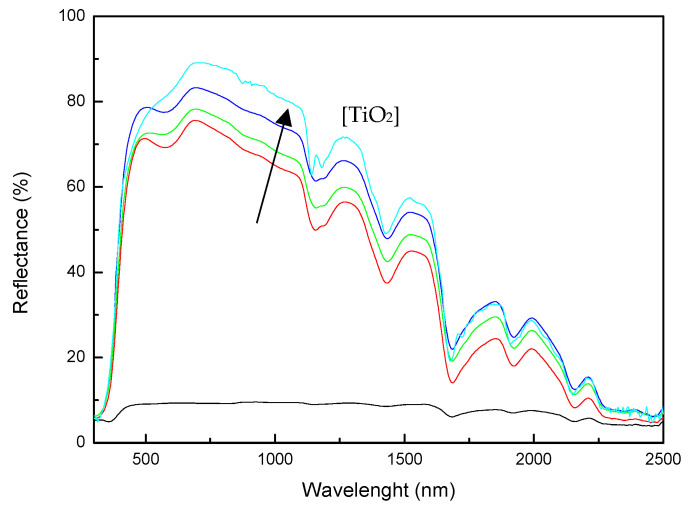
Reflectance spectra for (—) neat UP-MEKP system and systems with (—) 5, (—) 10, (—) 15 and (—) 20 wt%TiO_2_.

The total solar reflectance of the resins and nanocomposites were characterized by UV-Vis-NIR spectrophotometry, measuring the reflectance of the materials in the range of 300–2500 nm. The incorporation of the nanoparticles greatly increased the irradiated energy reflected by the materials, as can be seen in Figure 6. The incorporation of the nanoparticles increased the TSR value from <10 for the neat UP system to 70 for UP system modified with 20 wt% TiO_2_ (Table 2), due to the reflectance properties of the TiO_2_ nanoparticles and the white color of the modified resin [31]. The solar reflectance of modified resins is comparable to those of “cool coatings” reported in the literature [32,33,34]. These high-reflectance nanocomposites have the ability to reflect a significant part of near-infrared radiation, which can contribute to a reduction of the use of heating, ventilation, and air conditioning (HVAC) [33]. Moreover, the obtaining of a white system could avoid the use of gel coats during composite compounding or subsequent painting of cured composites, thus allowing the obtaining of a ready-to-use system.

**Figure 7:**
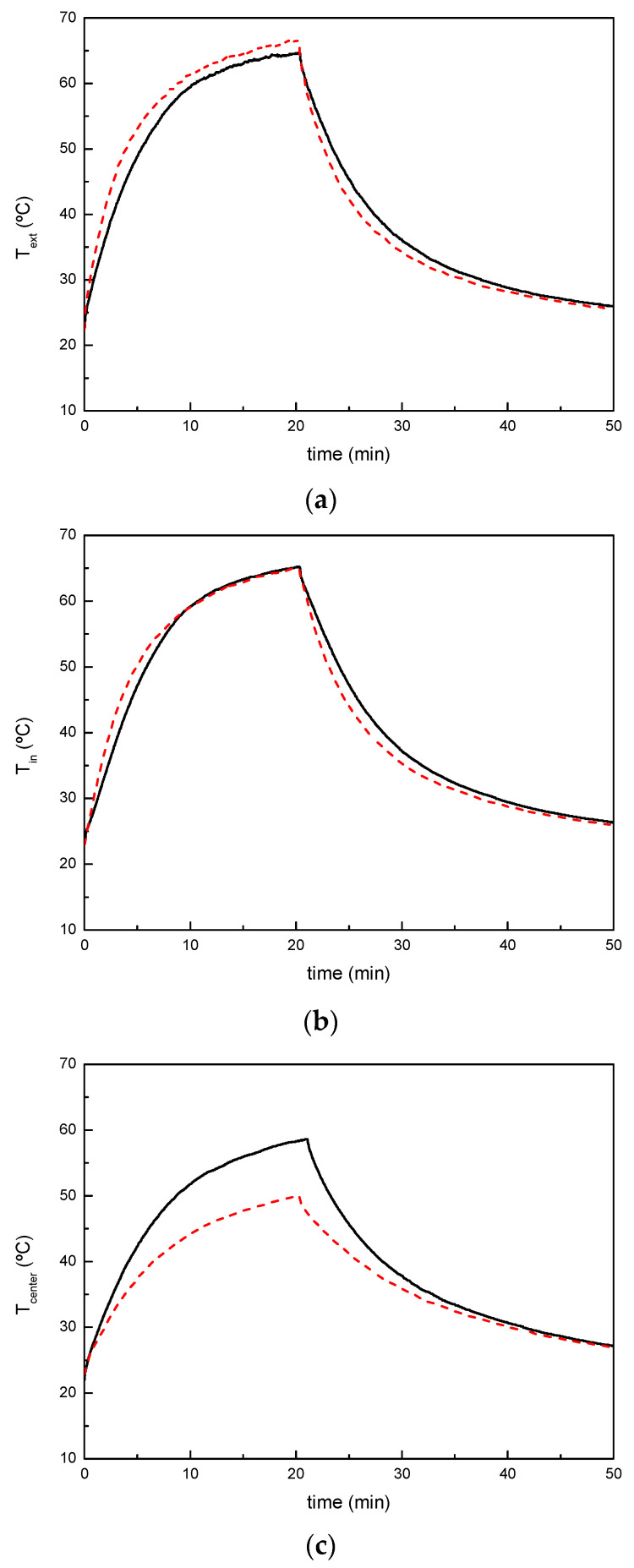
Thermal performance comparison between (—) neat UP system and (---) system modified with 20 wt.% TiO_2_: (**a**) sample external surface temperature; (**b**) sample inner surface temperature and (**c**) temperature at the center position in test box.

Thermal performance of nanocomposites was evaluated by exposing them to infrared light and analyzing the heat transfer through the composites when placed in the test box shown in Figure 2. Figure 7 shows the comparison of thermal performance between the unmodified and 20 wt% TiO_2_ modified systems. As shown in Figure 7a, the external surface temperature of the specimen corresponding to the system modified with 20 wt% TiO_2_ increases slightly faster than for the neat system and achieves slightly higher values, due to the higher conductivity of the modified systems. The thermal conductivity value increases from 0.46 ± 0.01 for the unmodified system to 0.6 W/mK ± 0.01 for the 20 wt% TiO_2_ modified system [35]. The internal surface temperature of the specimen corresponding to the modified system increases slightly faster than specimens of unmodified system, but the maximum achieved temperature is similar for both unmodified and modified systems (Figure 7b). Figure 7c illustrates the temperature histories in the center position of test box. It is clearly observed that the presence of the TiO_2_ nanoparticles reduces the temperature in the box. Even if the thermal conductivity of the resin can be slightly higher due to the presence of TiO_2_ nanoparticles, the high-infrared reflection capacity of the modified systems can reduce the heat transfer through the panel reducing the temperature of an insulated volume next to the panel, representing the indoor temperature in a vehicle, by 13 °C with respect to the use of the unmodified system.

### 3.3. Photocatalytic Properties

**Figure 8:**
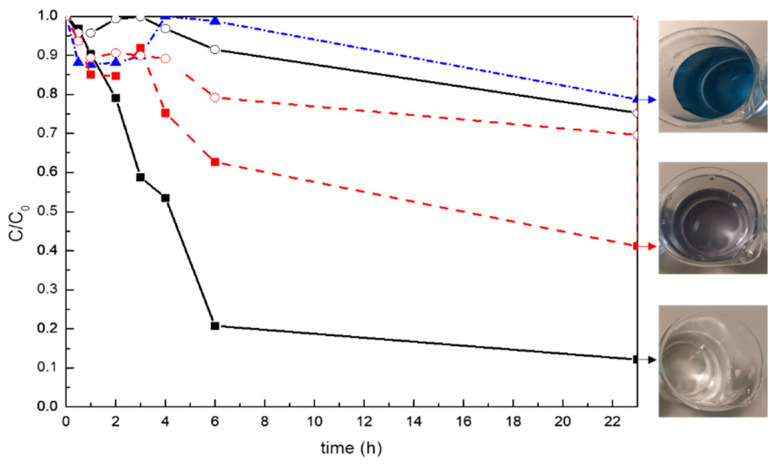
Photocatalytic degradation at 597 nm of (- - -) Remazol Black B solution and solutions in contact with (---) neat UP system and system modified with (—) 20 wt%TiO_2_. (■) tests carried out under UV radiation, and (○) test carried out in darkness. The degradation of Remazol Black B solution under UV light has been included for comparison (▲). Images of dye solution’s color after 23 h under UV radiation for Remazol Black B solution and solutions in contact with non-modified resin and 20 wt%TiO_2_ modified resin.

The photocatalytic degradation of Remazol Black B 3 mg/L by the modified composite was monitored by measuring the decrease of dye absorbance at 597 nm with respect to the irradiation time. Figure 8 shows the time evolution of the relative concentration of the dye C/C_0_, where C is the dye concentration at a given time and C_0_ is the initial dye concentration. Direct photolysis experiments were preliminarily run showing that Remazol Black B is stable over the irradiation time interval considered, degrading around 20% of the dye in 23 h. Adsorption of Remazol Black B on the surface of the modified resin was evaluated in darkness conditions. The behavior of a reference sample, the unsaturated polyester resin without nanoparticles, was also monitored under UV radiation and in darkness, as a reference.

The results showed a slight reduction in the relative concentration of solutions in contact with neat and 20 wt% TiO_2_ modified resin when they are not exposed to UV radiation, which was related to the partial adsorption of Remazol Black B on the surface of these specimens. This adsorption is similar for both materials after 23 h, as can also be observed in Table 3. 

**Figure 9:**
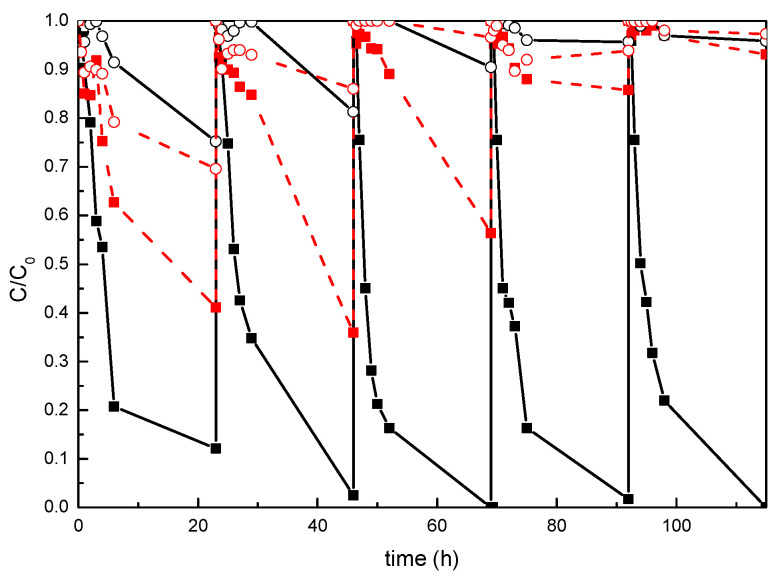
Photocatalytic degradation of Remazol Black B at 597 nm during the consecutive photocatalytic cycles for neat (---) UP system and system modified with (—) 20 wt% TiO_2_. (■) tests carried out under UV radiation, and (○) test carried out in darkness.

The analysis of these materials during five consecutive photocatalytic cycles, shown in Figure 9, indicated that this adsorption seems to be reduced with the cycles, as residues of Remazol Black B from the previous tests could remain on the surfaces of the specimens when they are subjected to the subsequent cycles, avoiding the adsorption of the dye. The last test carried out showed no reduction in relative concentration of the dye after 23 h. 

When these systems are radiated with UV light, a clear reduction in the color of solutions in contact with neat and 20 wt% TiO_2_ modified resin is observed. Surprisingly, the unmodified resin can degrade between 59% of the color of the solution after 23 h of UV exposure, which can be ascribed to the adsorption of the Remazol Black B, along with a partial photolysis of Remazol Black B under the UV radiation after 23 h [22] and also to the possible presence of some compounds in the commercial system that can interact with the dye. However, the reduction in dye concentration by photocatalysis was higher for the solution in contact with the 20 wt% TiO_2_ modified system, with 88% of the dye color being photocatalytically degraded within 23 h. The color variation of solutions in contact with the specimens of unsaturated polyester resin and resin modified with 20 wt% TiO_2_ nanoparticles after the exposition to UV light for 23 h was observed in Figure 8, highlighting the significant contribution of photocatalysis due to TiO_2_ nanoparticles to remove Remazol Black B from the solution. The kinetic constant (k) of both the photocatalytic reaction and the adsorption, was calculated by using a pseudo-first order kinetic model (dC/dt = kC), where C is concentration and k the apparent kinetic constant. Table 3 collects the reaction constants calculated through the fit of -ln(C/C_0_) vs. time curves, supporting the higher degradation rate of photocatalytic processes. After 5 cycles, the neat system presented reduced activity toward the dye, whereas the TiO_2_ modified system presented good stability and maintained its photocatalytic capability degrading 100% of the dye’s color after 23 h and five cycles, indicating the good long-term photocatalytic behavior and reusability of TiO_2_ modified resin. 

## 4. Conclusions

The reaction kinetics of an unsaturated polyester resin were studied by using DSC tests in both dynamic and isothermal modes. The reaction process was shown to be complex, as indicated by the presence of various peaks in dynamic thermograms. The presence of the nanoparticles shifts the thermograms toward higher temperatures and longer times, mainly due to the increase in the initial viscosity of the system and the dilution effect caused by the nanoparticles. The Tg^∞^ values of cured nanocomposites are smaller for systems with higher TiO_2_ content indicating a small steric hindrance effect due to the presence of the nanoparticles. The cured nanocomposites presented a homogeneous distribution of nanoparticles with a low degree of particle agglomeration. Concerning the thermal reflectance of the modified resins, the incorporation of TiO_2_ nanoparticles greatly increased the total solar reflectance values of the unsaturated resins from TSR < 10 for the neat UP system to 70 for UP system modified with 20 wt% TiO_2_, because of the reflectance properties of the TiO_2_ nanoparticles and of the change in color of the nanocomposites. The obtained white colored resins could allow the obtaining of ready to use composites. Moreover, the unsaturated polyester resin modified with 20 wt% TiO_2_ showed photocatalytic activity under UV radiation that remained with the cycles, degrading 100% of the color of the Remazol Black B solution after 23 h and five uses, whereas the unmodified resin displayed a reduced absorption activity under UV light over multiple cycles.

Summarizing, the incorporation of TiO_2_ nanoparticles to unsaturated resins leads to photocatalytic surfaces which can remove pollutants and maintain high solar reflectance of surfaces, which could contribute to a reduction in HVAC consumption, thus representing a possible solution for reducing global warming and pollutant emissions when they are incorporated as surface finishing for vehicles. 

## Figures and Tables

**Table 1:** Results for non-isothermal DSC tests for neat UP and systems with 5, 10, 15, and 20 wt% TiO_2_.

TiO_2_ wt%	T_onset_ (°C)	T_p1_ (°C)	T_p2_ (°C)	ΔH_T_ (J/g)	Tg^∞^ (°C)
0	50.0	60.0	100.0	291.0	66.8
5	56.0	64.5	105.0	328.0	67.2
10	68.0	75.0	113.0	279.5	66.0
15	71.0	79.5	117.0	256.5	64.2
20	75.0	86.0	116.5	231.0	64.7

**Table 2:** Reflectance and TSR values for the neat UP—MEKP system and systems with 5, 10, 15 and 20 wt% TiO_2_.

(wt%).	TSR (%)
0	8.8
5	61.1
10	64.1
15	69.9
20	72.6

**Table 3:** Remazol Black B color removal of solutions in contact with neat UP system and system modified with 20 wt% TiO_2_ after 23 h and rate constant (K) values for adsorption and photocatalytic processes.

System	Process	Cycle	Color Removal (%)	k (h^−1^)	R
neat UP	adsorption	1st	30.4	0.01364	0.82
		5rd	2.7	0.01240	0.72
neat UP	photocatalysis	1st	58.9	0.03673	0.90
		5rd	9.9	0.00257	0.76
UP + 20 wt%TiO_2_	adsorption	1st	24.7	0.01203	0.92
		5rd	4.1	0.00189	0.71
UP + 20 wt%TiO_2_	photocatalysis	1st	87.9	0.09223	0.76
		5rd	100	0.26496	0.97

## Data Availability

The raw/processed data required to reproduce these findings cannot be shared at this time as the data also form part of an ongoing study.
